# Differentiating sepsis from non-infective systemic inflammatory response syndrome: comparison between C-reactive protein and leptin

**DOI:** 10.1186/cc13404

**Published:** 2014-03-17

**Authors:** N Farag, K Taema, E Abdel-Latif, G Hamed

**Affiliations:** 1 Alsahel Teaching Hospital, Cairo, Egypt; 2 Cairo University, Cairo, Egypt

## Introduction

Differentiation of sepsis from non-infectious SIRS is important in improving sepsis outcome. We intended in this study to evaluate the role of serum leptin and to compare it with CRP in differentiating sepsis from non-infectious SIRS.

## Methods

We included 30 patients with SIRS. According to the presence or absence of infection, our patients were classified into the SIRS group and the sepsis group. Leptin and CRP were evaluated in all patients on admission, day 2 and day 4.

## Results

Our patients had mean age of 52.3 ± 18.6 years, 10 males (33.3%). There were no significant differences regarding baseline demographic and clinical data apart from blood pressures, which were lower in sepsis group. Serum leptin on day 2 only was higher in the sepsis group (44.2 ± 17.7 μg/l vs. 31.1 ± 2.1 μg/l, *P *= 0.008) with no difference on days 0 and 4 of admission. We detected a serum leptin level of 38.05 μg/l on day 2 to be 93% sensitive and 100% specific to diagnose sepsis. The three serum CRP levels were higher in the sepsis group compared with the SIRS group (61.2 ± 9 mg/l vs. 48.9 ± 7.1 mg/l, *P *< 0.001 on day 0, 71.5 ± 9.6 mg/l and 196.8 ± 39.8 mg/l in the sepsis group vs. 56.9 ± 8 mg/l and 73.7 ± 32.5 mg/l in the SIRS group for days 2 and 4 respectively, *P *< 0.001 for both). We found a CRP of 67.5 mg/l on day 2 having 87% sensitivity and 93% specificity for the diagnosis of sepsis.

## Conclusion

We concluded that although serum leptin may not be beneficial in early differentiation between sepsis and non-infectious SIRS on admission, it may be highly specific on the second day.

